# You Mate, I Mate: Macaque Females Synchronize Sex not Cycles

**DOI:** 10.1371/journal.pone.0026144

**Published:** 2011-10-12

**Authors:** Ines Fürtbauer, Roger Mundry, Michael Heistermann, Oliver Schülke, Julia Ostner

**Affiliations:** 1 Primate Social Evolution Group, Courant Research Centre Evolution of Social Behaviour, Georg-August-University Göttingen, Göttingen, Germany; 2 Integrative Primate Socio-Ecology Group, Max Planck Institute for Evolutionary Anthropology, Leipzig, Germany; 3 Reproductive Biology Unit, German Primate Center, Göttingen, Germany; 4 Max Planck Institute for Evolutionary Anthropology, Leipzig, Germany; 5 Courant Research Centre Evolution of Social Behaviour, Georg-August-University Göttingen, Göttingen, Germany; Yale University, United States of America

## Abstract

Extended female sexuality in species living in multimale-multifemale groups appears to enhance benefits from multiple males. Mating with many males, however, requires a low female monopolizability, which is affected by the spatiotemporal distribution of receptive females. Ovarian cycle synchrony potentially promotes overlapping receptivity if fertile and receptive periods are tightly linked. In primates, however, mating is often decoupled from hormonal control, hence reducing the need for synchronizing ovarian events. Here, we test the alternative hypothesis that females behaviorally coordinate their receptivity while simultaneously investigating ovarian cycle synchrony in wild, seasonal Assamese macaques (*Macaca assamensis*), a promiscuous species with extremely extended female sexuality. Using fecal hormone analysis to assess ovarian activity we show that fertile phases are randomly distributed, and that dyadic spatial proximity does not affect their distribution. We present evidence for mating synchrony, i.e., the occurrence of the females' receptivity was significantly associated with the proportion of other females mating on a given day. Our results suggest social facilitation of mating synchrony, which explains (i) the high number of simultaneously receptive females, and (ii) the low male mating skew in this species. Active mating synchronization may serve to enhance the benefits of extended female sexuality, and may proximately explain its patterning and maintenance.

## Introduction

Extended female sexuality (i.e. non-conceptive receptivity) has been reported for numerous vertebrate and invertebrate species and is most likely part of a female strategy to obtain male-delivered benefits (e.g. [1]–[7]). Many examples come from pair-living species including birds and mammals (e.g. [5], [7], [8]). Here, extended sexuality appears to predominantly function to gain material assistance delivered by primary partners (e.g. [4], [5]). Conversely, in mammals with multimale-multifemale social organization, such as many non-human primates, extended female sexuality often co-occurs with promiscuity, and thus, has been argued to enhance benefits from multiple males, namely to refrain from infanticide or to provide care for future infants (e.g. [2], [3], [6], [9]–[12]).

Females in multimale-multifemale groups, however, usually face the problem of being monopolized by a single (dominant) male (e.g. [11]–[13]). Thus, for extended sexuality to be effective, i.e. to enhance polyandrous mating, at least some degree of female behavioral freedom is required which can be achieved by increasing the number of simultaneously sexually active females. Female reproductive synchrony, i.e. the spatiotemporal clustering of receptive females, limits the degree to which females can be monopolized by a single male [14]–[22], hence enabling them to mate with multiple males and also to exert alternative mate choices (e.g. [3], [6], [23], [24]).

The temporal coordination of reproductive activity can be facilitated by seasonal breeding (likely to be regulated through external cues; [25], [26]) because several females may get into breeding condition during a short period of time [15], [22], or it can be achieved through active synchronization (see below). This may sometimes make it difficult to disentangle the potential ecological and social factors underlying reproductive synchrony, in particular in highly seasonal species (e.g. [18], [27]).

Studies focusing on mammalian reproductive synchrony are generally based on the assumption that females achieve synchrony by synchronizing (and asynchrony by desynchronizing) their ovarian cycles (e.g. [27]–[37]). Studies on rodents revealed mixed evidence for ovarian cycle synchrony (e.g. *Rattus norvegicus*: [33], [38]; *Mesocricetus auratus*: [28], [30]). Similarly, in primates, the results remain controversial and inconclusive. Apart from a few studies (*Homo sapiens*: [32]; *Pan troglodytes*: [37]; *Leonthopithecus rosalia*: [29]; but see [39], [40]) most investigations found no evidence for ovarian cycle synchrony (e.g. *Papio hamadryas*: [36]; *Homo sapiens*: [41]; *Mandrillus sphinx*: [35]; *Leontopithecus rosalia*: [34]) or report asynchrony (e.g. *Papio hamadryas*: [42]; *Pan troglodytes*: [31]; *Lemur catta*: [27]).

Several primate-typical reproductive features may potentially explain the lack of ovarian cycle synchrony observed in most species: First, and in contrast to most mammals (e.g. [26]), female receptivity in primates is usually decoupled from hormonal control (e.g. [2], [43], [44]). Second, most primates exhibit extended mating periods within ovarian cycles (some Old World monkeys mate throughout their complete cycle), a phenomenon that has been attributed to prolonged follicular phases [11], [45]. Third, primate females often show an increased number of cycles to conception, extending their total mating period (e.g. [46]), hence increasing the chance to overlap in mating activity with other females. Fourth, male knowledge about female fertility status usually is imperfect (e.g. [10], [47]). In general, the “need” for ovarian cycle synchrony in order to reduce male monopolization potential should decrease with (i) the degree of emancipation of sexual behavior from hormonal control, (ii) increasing length of the mating period, and (iii) increasing unpredictability of fertility (note that these factors are not independent). To illustrate an extreme: in species where fertility is undisclosed to males, and females potentially mate throughout and beyond their cycles, synchronizing ovarian events becomes unnecessary to achieve overlapping receptive periods. Furthermore, ovarian cycle synchrony cannot explain synchronous receptivity during acyclic stages (e.g. pregnancy).

Here, we focus on an alternative, non-mutually exclusive, hypothesis that females synchronize their mating activity. Assuming that male monopolization potential is affected by the spatiotemporal distribution of receptive females (e.g. [14]–[22]), and that mating is decoupled from hormonal control, females may mate non-randomly with respect to time, in order to avoid male monopolization. On a proximate level, and in contrast to ovarian cycle synchrony which is assumed to be regulated by pheromonal cues (e.g. rats: [48]; humans: [49], [50]), mating synchrony may be achieved through behavioral coordination [51], i.e. females may respond to the mating activity of other females in the group. In other words, the probability of a given female to copulate may be affected by the number of other females mating.

To our knowledge, no study has yet attempted to investigate whether females synchronize their mating activity independent of ovarian cycle synchrony. Here, we use hormone and mating data obtained from wild female Assamese macaques (*Macaca assamensis*) to test this hypothesis. Using permutation procedures and Linear Mixed Effects Models we investigate whether females synchronize their mating activity. We simultaneously examine whether female fertile phases are significantly more synchronous or asynchronous than expected from a random distribution using permutation procedures. Also, we investigate whether dyadic spatial proximity affects the temporal distribution of fertile phases, to test the hypothesis that those females who spend more time in close proximity cycle more closely together (e.g. see [48], [49]).

Assamese macaques breed seasonally, and females mostly conceive in their first ovarian cycle [52] with fertile phases overlapping to some extent [53]. Females exhibit no apparent coordination between receptive and fertile periods as they become sexually receptive (i.e. sexually active) in unison (up to 3 months before the onset of cyclic ovarian activity) and mate, at low daily frequencies, throughout the 4-month mating season and even into pregnancy [53]. As a consequence, on nearly 90% of mating season days there is more than one female receptive. The extreme extended female sexuality together with concealed fertility [53] diminishes male monopolization potential which is reflected in an unusually low alpha male mating skew (17.5%; [54]). Despite a high degree of promiscuity (i.e. females mate with virtually all males) females exhibit non-dominance based mating biases towards different (high- and low-ranking) male individuals [53]. Only top ranking males engage in long sexual consortships, not linked to female fertile phases [53], [54]. The strong seasonality of the species may account for some overlap in both fertility and receptivity. The extreme non-conceptive mating activity beyond ovarian cyclicity (see [53]), however, is puzzling and making the setting ideal to test whether females behaviorally coordinate their mating activity.

## Methods

This study was carried out in the field with wild monkeys and was completely non-invasive. Approval and permission to conduct research was granted by the authorities of Thailand (permit no. 0004.3/3618), and all research was undertaken in strict accordance with the ABS/ASAB guidelines for the ethical treatment of animals in research, the recommendations of the Weatherall report on the use of non-human primates in research, and the laws set forth by the National Research Council of Thailand and the regulations of the Department of National Parks, Wildlife and Plant Conservation, Bangkok, as well as the guidelines of the involved institutes.

### Subjects

During two consecutive mating seasons (MS) behavioral and hormone data were obtained from a wild group of Assamese macaques at the Phu Khieo Wildlife Sanctuary (157,300 ha, 16°5′-35′N, 101°20′-55′E, 300-1,300 m a.s.l.), north-eastern Thailand. During the MS 07/08 (Oct 1^st^-Jan 31^st^) the study group comprised 53 individuals including 13 males and 12 adult females, seven of which conceived and data are presented for. During the MS 08/09 (Oct 1^st^-Feb 13^th^) the group consisted of 55 individuals including 15 males and 14 adult females, ten of which conceived and eight of which data are presented for (two females conceived unexpectedly, hence had not been sampled). No subject was included twice in the study (i.e. n = 15 females).

### Behavioral data

The study group was followed from dawn to dusk (2,837 contact hrs; 11.1±0.7 contact hrs/day) throughout the two mating seasons combining *ad libitum* and focal observations. We recorded social and sexual behaviors (for details on female sexuality see [53]), and conducted proximity scans (n = 5627) every ten minutes during focal observations by noting all females within 5m of the focal female. In order to control for rank effects, female dominance rank was established based on the exchange of clear submissive signals, i.e. “silent bared teeth” [55] and “make room” [56]. Both mating seasons were treated separately because one female died in June 2008, and three primiparous females were added to the data set in the MS 08/09. Female dominance hierarchy was assessed using the I&SI method as implemented in MATMAN™ 1.1.4 (Noldus 2003). We then standardized ranks to a range from 0 (lowest ranking) to 1 (highest ranking), and with the females considered in the study evenly spaced between these two values.

### Assessment of fertile phases

Fecal hormone analysis to assess ovulation and fertile periods has been described in detail by Fürtbauer et al. [52], [53]. In brief, we collected on average±sd 4.6±0.5 samples per week from each study female. Following hormone extraction from freeze-dried samples, extracts were measured for concentrations of progestogens (20α-dihydroprogesterone; 20α-OHP) using a validated microtiterplate enzyme immunoassay (EIA; [52]). Sensitivity of the assay at 90% binding was 1.5 pg. Intra- and interassay coefficients of variation, calculated from replicate determinations of high- and low-value quality controls were 7.5% and 13.1% (high) and 9.2% and 16.7% (low).

As described in Fürtbauer et al. [52], the timing of ovulation was determined by using the defined post-ovulatory rise in fecal progestogen levels, taking into account the fecal excretion time lag. Day -3 relative to the day of the defined progestogen increase (day 0) was considered as the most likely day of ovulation. The fertile phase was defined as a 5-day period including days -2 and -3 (relative to day 0) plus the three preceding days (see [53]). For two females the exact timing of the fertile phase could not be determined but could be narrowed down to the first half of February 2009.

### Ovarian cycle synchrony

To test for synchrony of fertile periods we used the approach of Matsumoto-Oda et al., [31]. We measured the Synchrony-Index (SI) as described therein and tested its significance based on 1,000 permutations into which we included the original data as one permutation. Units of permutation were the fertile periods and the intervals between them (permuted separately). Other than in Matsumoto-Oda et al. [31] we imposed a restriction on the permutation algorithm which ensured that the total duration of the period lasting from the beginning of the first to the end of the last fertile period was kept constant. In case a female went through two fertile periods (n = 3 females, MS 07/08; see [52]) we kept the time interval between them constant and just permuted the two fertile periods. Since synchrony could in principle be smaller as well as larger (i.e. asynchrony versus synchrony) we determined two one-tailed p-values. In the MS 08/09 the precise timing of the fertile periods for 2 females was not known (see above). Hence, we constructed data sets representing all possible combinations of timings of the fertile periods of these two females and tested each of them using the approach described above. For these we report the average p-value. Permutation tests were calculated using a program written by R.M. (we did not use a linear model for this analysis because fertile periods last 5 continuous days, hence making the occurrence of fertile and non-fertile days within periods clearly non-independent).

To test whether close spatial proximity between females affected the timing of their fertile phases (see [48], [49]), we first determined for each dyad the proportion of scans at which the two females were no more than five meters apart. Furthermore, we determined for each dyad the absolute number of days elapsed between the onsets of their fertile phases. We then correlated the two matrices using a Mantel test [57]. This test was exact (i.e. enumerating all possible permutations of the data) and based on Spearman's rho as the test statistic. For the MS 08/09 where the exact onset of fertile phases could not be determined for two females, we used the approach described above (averaging the result for all possible combinations of onset days). Mantel tests were calculated using a program written by R.M.

### Mating synchrony

To test whether females synchronized days on which they copulated we used the following approach: First, we calculated for each female and day the proportion of the other females which copulated that day. We then used a Generalized Linear Mixed Model (GLMM; [58]) to test whether the probability of a given female to copulate (response variable: yes/no) on a given day was influenced by the proportion of other females copulating that day. Besides the proportion of females copulating we also included the conception status (pre- and post-conception), its interaction with the former variable, and the females' rank as fixed effects. In addition, we included female ID and MS (07/08 and 08/09) as random effects.

The response variable in the model was likely to show temporal autocorrelation unexplained by the fixed effects included. Thus, the assumption of independent residuals was likely to be violated (i.e. neighboring residuals being more similar than more distant ones) devaluating the reliability of the model. Hence, we decided to explicitly incorporate temporal autocorrelation into the model using the following approach: We first ran the model with all fixed and random effects included and derived the residuals from it. Then, for each data point we calculated an ‘autocorrelation term’ as the average of the residuals of all other data points of the same female with the contribution of the residuals being weighted by their time lag to the particular data point. The weight followed a normal distribution, with its standard deviation determined by minimizing the AIC [59] of the GLMM including the autocorrelation term as an additional fixed effect.

Regarding significance testing we first determined the significance of the full model (including all fixed effects, the interaction, the autocorrelation term and random effects) as compared to the corresponding null model (including only the autocorrelation term and the random effects) using a likelihood ratio test [60]. Only if this revealed significance we considered the significance of the individual predictors. P-values of main effects we considered only if they were not included in a significant interaction.

We calculated the GLMM in R (version 2.11.1, [61]) using the function lmer of the R package lme4 [62]. GLMM's were fitted with binomial error structure and logit link function and likelihood ratio tests were calculated using the R function anova. To enhance the reliability of this likelihood ratio test we used maximum likelihood estimation in the mixed model (argument REML of the function lmer set to FALSE). Significance of the individual fixed effects was determined based on the z- and p-values provided by lmer. The autocorrelation term was calculated using a function written by R.M. and the minimization of the AIC to find the best fitting standard deviation of the weight function for the autocorrelation term was done using the R function optimize.

Additionally, we used a permutation procedure similar to that described for ovarian cycle synchrony (see above). We did this because the assessment of P-values for fixed effects can be unreliable in Mixed Models [63]. We first measured the variance in mating synchrony as described in Matsumoto-Oda et al. [31] and then used the following approach: we first identified, separately for each female, sections of consecutive days all of which comprising at least one copulation, and sections of consecutive days none of which comprising at least one copulation. We then permuted these sections, separately for sections with and without copulations. In addition, we permuted data only for days between the first and the last day with copulation. Since there were two days without observations in the MS 07/08 and one day without observations in the MS 08/09, we subdivided the study period into three phases in the MS 07/08 and two phases in the MS 08/09, respectively, and permuted data only within these phases. We determined the P-value as the proportion of permutations revealing variance in synchrony being at least as large as that of the original data. We used 1,000 permutations and the original data were included as one permutation.

## Results

### Ovarian cycle synchrony

We investigated patterns of ovarian cycle synchrony during two mating seasons and found no evidence of synchrony or asynchrony of female conceptive fertile phases (5 days; hormonally determined; MS 07/08; SI = 0.187, p_(syn)_ = 0.102, p_(asyn)_ = 0.939; MS 08/09: SI = 0.118, p_(syn)_ = 0.455, p_(asyn)_ = 0.640; no error level correction applied). Because in the MS 07/08 three females conceived during their second cycle (all other females in the same year and in the MS 08/09 conceived in their first cycle), we ran the permutation procedure with including the three non-conception cycles, and achieved similar non-significant results (SI = 0.162, p_(syn)_ = 0.2, p_(asyn)_ = 0.834). Dyadic spatial proximity of females was not correlated with the interval between the onsets of fertile periods (Mantel test, MS 07/08: r_S_ =  −0.04, n = 7 females, p = 0.84; MS 08/09, average of 48 tests: r_S_ = 0.22, n = 8 females; p = 0.24).

### Mating synchrony

A GLMM revealed evidence that females synchronized their mating activity. We found an overall significant effect of the entire set of predictor variables included in the model (likelihood-ratio test comparing the fit of the full with that of the null model comprising only the random effects: χ^2^ = 22.30, df = 4, N = 1927 days and 15 females, p<0.001). The probability of a given female to copulate (response variable: yes/no) on a given day clearly increased with the proportion of other females copulating that same day (Table 1). In addition, higher ranking females were more likely to copulate. Female conception status (i.e. pre- or post-conception) had no significant effect on the probability to copulate.

**Table 1:** Factors influencing the probability of a given female to copulate on a given day (binary variable).

predictor variable	estimate ± SE	z value	p
Intercept	−1.14±0.16	−7.18	<0.001
Other females copulating	0.64±0.24	2.71	0.007
Conception status	0.17±0.12	1.38	0.17
Dominance rank	0.87±0.20	4.47	<0.001
Autocorrelation term	2.00±0.17	11.45	<0.001

Predictor variables: Proportion of other females copulating, conception status (pre- or postconception), female dominance rank (standardized across the two mating seasons), and autocorrelation term.

Female ID (n = 15) and season (n = 2) were included as random factors. The interaction between the number of other females copulating and conception status was not significant (estimate±SE = -0.05±0.51, z = -0.10, P = 0.92). The numbers presented in the table are from a model not comprising this interaction.

Because assessing the significance of fixed effects in the framework of Mixed Models is associated with some uncertainty [63], we backed up our conclusions with a permutation test as applied for the synchrony of the fertile phases (see above). We obtained matching results in that sections of consecutive mating days (see methods for details) were more synchronous than expected by chance (SI = 0.175, p = 0.002).

## Discussion

Our data show that although in Assamese macaques female fertile phases partly overlap (see [53], Fig. 4), those females who spent more time in close proximity did not cycle more closely together (pheromonal hypothesis; [48], [49]; but see, e.g. mandrills: [35], chimps: [64]; mouse lemurs: [65]), and that the observed ovarian cycle overlap did not deviate from random expectations. In contrast, we provide evidence for significant synchrony of mating activity.

The result that females did not synchronize their cycles is not surprising because specific reproductive and life-history traits may “prevent” females from synchronizing their ovarian cycles. First, Assamese macaque females are anovulatory during the non-mating season and mostly conceive during their first ovulatory cycle within the mating season [52]. ‘Active’ ovarian cycle synchronization, however, is likely to require several cycles (e.g. [32]). A recent study on mandrills (*Mandrillus sphinx*), for example, found evidence for significant female cycle synchrony in only one out of ten years, the year with most cycles (overall and per female) recorded, suggesting a link between ovarian cycle synchrony and the number of cycles to conception [35]. Second, as shown recently for Assamese macaques, early conception within the mating season may allow for a 1-year inter-birth interval (IBI) whereas females conceiving late in the season are more likely to conceive their next infant after two years [52]. Any shifting of ovarian cycles towards the end of the mating season, for the benefit of ovarian cycle synchrony, may have to be paid with a 2-year instead of a 1-year IBI, and thus considerably affects female lifetime reproductive success, and should not be selected for. In line with this, the period between parturition and consecutive conception in Assamese macaque females with 1-year IBIs is relatively constant (265 to 290 days; [52]), indicating that females are impregnated as soon as they resume cycling (female primates need to attain a critical body weight to resume cycling; e.g. [66], [67]). Third, the main hypothesis to explain ovarian cycle synchrony is to increase the number of simultaneously receptive females in order to decrease male monopolization potential and to mate with multiple males (e.g. [14]-[22]). In Assamese macaques, however, fertility is undisclosed to males, and females mate rather continuously throughout the 4-month mating season, i.e. during acyclic, cyclic, and pregnant stages [53]. So, females do not need to synchronize their fertile periods because males appear to be unable to recognize them, and because mating is largely decoupled from hormonal control.

The alternative hypothesis tested here is that females actively synchronize their mating not their ovarian activity. We found that the occurrence of a female's receptivity on a given day was significantly associated with the proportion of other females mating that day. Female Assamese macaques start being sexually active in unison with some females mating up to three months before they experience their first ovulatory cycle (see [53], Fig.1). Our results not only explain the high number of simultaneously receptive females not due to ovarian cycle synchrony and environmental seasonality, but also offer a possible proximate explanation for the patterning and maintenance of extended sexuality as described in the following scenario: In Assamese macaques, conceptions are spread over the mating season, with few conceptions occurring in October, i.e. at the beginning of the mating season [52]. The onset of the mating season is likely to be triggered by external cues (e.g. photoperiod; [25], [26]) which induce the onset of ovulatory and sexual activity in a few females, namely those who conceived their last infant two years ago (see above). Other females who resume cycling later in the season start being receptive in response to the mating of these females. Thus, receptivity is socially (behaviourally and not via pheromones) mediated and induced before the onset of ovulatory activity and, furthermore, maintained after conception, i.e. during pregnancy. In Assamese macaques, 70% of copulations are female-initiated [53] (and females very rarely refuse copulations; IF, pers. obs.), which supports the active nature of the observed female mating synchrony.

Social facilitation of sexual behavior has already been proposed for patas monkeys (*Erythrocebus patas*; [68]). A study on rhesus macaques (*Macaca mulatta*) has shown that not only male sexual behavior increased due to the presence of females which were experimentally brought into sexual receptivity during the non-mating season but also untreated females exhibited copulatory behavior [69]. In blue monkeys (*Cercopithecus mitis stuhlmanni*), the simultaneous onset of receptivity may also be socially mediated; however, no endocrine data are available in order to rule out ovarian cycle synchrony [70]. The mating patterns of vervet monkeys (*Cercopithecus aethiops*; see [71], [72]) and Tibetan macaques (*Macaca thibetana*; see [73]) both closely resembling that of Assamese macaques, suggest a behaviorally coordinated mating activity. It would be rewarding to test whether female receptivity is more synchronous than random in the species mentioned above in order to evaluate the general applicability of the mating-synchrony-hypothesis. Also, the discrepancy between observed and expected female mating overlap in some primate species (see [19], [74]), may, given our results, be explained by active mating and not necessarily ovarian cycle synchrony (which anyway is disputed in primates; e.g. [39]). Theoretical support for socially induced mating behavior comes from a recent model on the evolution of increased female sexuality which indicates that sexual behavior outside fertile periods can occur as soon as some fertile females appear in the population [5].

On an ultimate level, although not tested here, behavioral coordination [51] of sexual activity may have different functions. The two main potential explanations, depending, at least partly, on the social system of the species, relate to (1) female-female reproductive competition and (2) intersexual conflict over paternity concentration. In uni-male groups of western lowland gorillas (*Gorilla gorilla*), for example, females synchronize post-conception copulations to occur when other females mate, which has been linked to female mating competition [75], [76]. In contrast, in species with multimale-multifemale social organization, we propose that active mating synchrony may be an effective female strategy whenever paternity concentration in the dominant male is less important than paternity dilution among many males (e.g. inside take-over species; see [77]), or when females adopt an alternative mate choice strategy not based on male dominance rank (e.g. MHC-associated mating; [78], [79]). In Assamese macaques, females are highly promiscuous (i.e. they mate with virtually all males) while at the same time expressing non-dominance based mating biases (towards different males), and mating repeatedly with their ‘primary partner’ (for details see [53]). Furthermore, in this species, females rarely interfere in copulations of other females (IF, pers. obs.), indicative of female-female competition being an unlikely explanation for the observed mating synchrony. Active mating synchrony, i.e. great flexibility in sexual behavior, throughout the mating season (i.e. pre- and post-conception) appears to diminish largely male monopolization potential and reproductive skew in Assamese macaques (17.5% alpha male mating share; [54]), which enables females to exert their reproductive strategy, i.e. creating differentiated mating relationships within a promiscuous mating system (see [53]).

Finally, we call for the use of consistent terminology. Irrespective of conflicting evidence for synchrony in the context of animal reproduction, numerous terms (ovarian-, menstrual-, estrous-, cycle-, female-, receptive-, reproductive-, breeding-, and mating synchrony) have been inconsistently used between and within studies in order to describe any overlap in female reproductive events. Hence, we explicitly stress the differentiation of physiological and behavioral reproductive synchrony given our finding that mating synchrony can occur in the absence of ovarian cycle synchrony.
